# The relationship between payer type and lipid outcomes in response to clinical lifestyle interventions in youth with dyslipidemia

**DOI:** 10.1186/s12887-019-1593-5

**Published:** 2019-07-02

**Authors:** Jacob C. Hartz, Elizabeth Yellen, Annette Baker, Justin Zachariah, Heather Ryan, S. Skylar Griggs, Nirav K Desai, Ravi Yanumula, Samuel Vinci, Caroline Brantley, Jennifer Bachman, Ellen McAuliffe, Kimberlee Gauvreau, Michael Mendelson, Sarah de Ferranti

**Affiliations:** 1 Boston Children’s Hospital, 300 Longwood Ave, Boston, MA 02115 USA; 2 Boston Medical Center, 850 Harrison Ave., 6th floor, Boston, MA 02118 USA; 3 Texas Children’s Hospital Main Campus, 6651 Main Street, Legacy Tower, 21st Floor, Houston, TX 77030 USA; 4 School of Nursing, University of Massachusetts-Boston, 100 William T. Morrissey Blvd, Boston, MA 0212 USA; 5 School of Public Health, University of Washington, 1959 NE Pacific St, Seattle, WA 98195 USA

**Keywords:** Children and adolescents, Dyslipidemia, Obesity, Health insurance, Lifestyle interventions

## Abstract

**Background:**

Payer-type (government-sponsored health coverage versus private health insurance) has been shown to influence a variety of cardiovascular disease outcomes in adults. However, it is unclear if the payer-type impacts the response to a lifestyle intervention in children with dyslipidemia.

**Methods:**

We analyzed data prospectively collected from patients under the age of 25 years who were referred to a large regional preventive cardiology clinic from 2010 to 2016 in Massachusetts. We compared baseline high density lipoprotein cholesterol (HDL-C), triglycerides (TG), non-HDL-C, and low density lipoprotein cholesterol (LDL-C) by payer-type. Further, we analyzed the change in lipid values in response to a clinic-based multidisciplinary intervention over a nearly six-year period by payer-type with multi-variable adjusted linear regression models. We also tested for effect modifications by age, sex, race, and body mass index (BMI) category.

**Results:**

Of the 1739 eligible patients (mean age 13 years, 52% female, 60% overweight and obese, 59% White), we found that patients with government-sponsored coverage (*n* = 354, 20%) presented to referral lipid clinic with lower HDL-C (− 3.5 mg/dL [1.0], *p* < 0.001) and higher natural log-transformed TG (+ 0.14 [0.04], *p* < 0.001) as compared to those with private insurance; however, the association was attenuated to the null after additionally adjusting for BMI category (− 1.1 [0.9], *p* = 0.13, and + 0.05 [0.04], *p* = 0.2 for HDL-C and natural log-transformed TG, respectively). We found no difference in baseline LDL-C between payer-types (+ 3.4 mg/dL [3.0], *p* = 0.3). However, longitudinally, we found patients with private insurance and a self-reported race of White to have a clinically meaningful additional improvement in LDL-C, decreasing 12.8 (5.5) mg/dL (*p* = 0.02) between baseline and first follow-up, as compared to White patients with government-sponsored health coverage, after adjusting for age, sex, time between visits, and baseline LDL-C.

**Conclusions:**

Our results suggest that youth with government-sponsored coverage are referred with poorer lipid profiles than those with private insurance, although this is largely explained by higher rates of overweight and obesity in the government-sponsored health coverage group. White patients with private insurance had substantially better improvement in LDL-C longitudinally, suggesting that higher socioeconomic status facilitates improvement in LDL-C, but is less beneficial for HDL-C and triglyceride levels.

## Background

Pediatric dyslipidemias are relatively common; depending on the population sample and thresholds used, up to 20% of children and adolescents have abnormal levels of low-density lipoprotein cholesterol (LDL-C), high-density lipoprotein cholesterol (HDL-C), or triglyceride concentrations [1–5]. Without intervention, childhood dyslipidemia often persists into adulthood, increasing the risk for premature cardiovascular disease (CVD) [6–9]. The cornerstone of managing pediatric lipid abnormalities is lifestyle modification counseling to optimize physical activity and the diet [10]. However, opportunities for improvements in lifestyle may be constrained by limited resources in families who qualify for government-sponsored health coverage as eligibility is typically based on income thresholds. There is limited information on the relationship between payer-type (government-sponsored health coverage versus private health insurance) and dyslipidemias among children and adolescents, especially as it relates to patient responses to intensive lifestyle counseling [11–14].

Health insurance status is associated with health outcomes across a wide range of diseases [15]. In adults, insurance coverage has been shown to correlate with CVD risk factors, including an atherogenic lipid profile and more frequent CVD events [16, 17]. However, these studies may be biased when the disparities in socioeconomic status (SES) are a result of, rather than a contributing factor to, the medical condition. For example, a debilitating illness will often limit earning potential and result in lower SES and need for government-sponsored health coverage. Children provide an ideal cohort to study the role of payer-type on dyslipidemia outcomes as the child’s dyslipidemia status is unlikely to impact their families’ income and eligibility for government-sponsored coverage.

To develop and implement clinical lifestyle interventions effectively and efficiently, it is important to understand the contribution of the payer-type on pediatric dyslipidemia and response to current treatment approaches. However, there is limited information on the relationship between payer-type and change in lipid values among children and adolescents undergoing clinical lifestyle counselling [11–14]. Even in adults it is not always only limited financial resources that affect the lifestyle improvements but other factors as well, (e.g. inadequate perception of CVD risk factors) [18]. We hypothesize that children with dyslipidemia and government-sponsored health coverage will not respond as well to clinical-based lifestyle counseling interventions compared to children with private insurance. To address this gap in knowledge, we examined standardized data prospectively collected as part of a quality improvement (QI) effort conducted in a large, multi-disciplinary pediatric preventive cardiology clinic to determine whether baseline dyslipidemia and response to intensive lifestyle counseling differed between those with government-sponsored health coverage versus private insurance.

## Methods

### Study population

As part of an ongoing QI project, we prospectively collected clinical and demographic data from patients under the age of 25 years who were assessed in the Preventive Cardiology Program at a large regional medical center from September 1, 2010 to March 31, 2016. Patients were eligible for this study if they had an initial visit to the Preventive Cardiology Program during the observation period with a lipid profile that included a total cholesterol, triglyceride level, HDL-C, and LDL-C, regardless of the reason for referral. For example, patients who were referred to the Preventive Cardiology Program for hypertension, but obtained a lipid profile as part of the visit. Patients were excluded if they were prescribed lipid-lowering medications during follow up at any time during the observation period, if they were over the age of 25 years, or if they did not have a baseline visit in the observation period. This study was approved by the research ethics board with a waiver of individual participant consent.

### Study design

The QI initiative was implemented for all children and adolescents seen for a lipid disorder in a Preventive Cardiology Program as part of a large, regional medical system starting September 1, 2010. This initiative provided management suggestions and prospectively collected clinical data via the use of a Standardized Clinical Assessment and Management Plan (SCAMP). Providers (physicians, nurse practitioners, and nurses) completed the standardized forms for each patient encounter and when interval laboratory measurements were obtained. In addition to collecting pertinent health information, the SCAMP suggested management by way of treatment algorithms generally consistent with the 2011 US National Heart, Lung, and Blood Institute Pediatric Integrated CVD Guidelines [10]. Patients were counseled on heart healthy lifestyle behaviors based on the 2011 Guidelines; patients visited with a registered dietician and a nurse practitioner or physician. The intervention included nutrition and physical activity counseling tailored for the type of lipid disorder. For example, for those patients with high triglycerides, the focus was on reducing intake of simple sugars and replacing them with vegetables and whole grains. In contrast, for patients with elevated LDL-C, the focus was more on reducing saturated fat and increasing dietary fiber intake in the form of fruits, vegetables and whole grains. Physical activity counseling focused on stepwise increases in activity to target a minimum of 5 h of moderate-to-vigorous physical activity per week. Patients had the opportunity to meet with a registered dietician at each visit. In addition to in-person counselling, patients were provided with printed educational materials. After three visits, additional visits did not necessarily include a visit with a dietician. Clinical information was extracted from this QI dataset and supplemented with information from the electronic health record (EHR).

### Payer-type

The participant’s payer-type was determined for the purposes of this investigation from the EHR at the initial clinic visit and was held constant during all longitudinal analyses. Government-sponsored coverage was defined as having any coverage through Medicaid, the State Children’s Health Insurance Program, or Medicare. Private coverage was defined as having health insurance that was not provided through government-sponsored coverage and also included international patients. No patients were identified as uninsured in our study. Out of state patients had insurance coverage by their respective state and were classified as private or government-sponsored in a similar fashion. Payer-type was present on administrative paperwork at each visit.

### Anthropometric and clinical assessments

Weight was recorded by a trained clinical provider via a standing scale (Scale-Tronix Stand-on Scale, Scale-Tronix, White Plains, NY) to the nearest 0.1 kg with patients in their own clothing without a jacket or shoes. However, it was not possible to know if a patient’s weight was measured on the same scale at each visit. Height was measured with a vertical stadiometer in patients without shoes to the nearest 0.1 cm. Body mass index (BMI) was calculated as weight in kilograms divided by height in centimeters squared. BMI percentiles and Z-scores were generated using the Centers for Disease Control (CDC) 2000 reference tables, as implemented in CDC EpiInfo version 7.

### Laboratory assessments

Lipid measurements were obtained from peripheral blood samples, generally after an 8-h fast. Measurements were made using standard enzymatic assays. Total cholesterol, HDL-C, and triglycerides were measured and LDL-C was calculated according to the Friedewald equation [19]. Non-HDL-C, a surrogate for all atherogenic Apolipoprotein B containing particles, was determines as total cholesterol minus HDL-C. If triglycerides were ≥ 400 mg/dL (4.52 mmol/L), a direct LDL-C was obtained in most cases.

### Statistical analysis

We generated summary statistics including counts, percentages, means and medians, as appropriate for distribution, to describe categorical and continuous study variables. Baseline characteristics were compared between payer-type with t-tests or chi-squared tests for continuous and categorical data, respectively. The triglyceride level was natural log transformed due to non-normal distribution. We defined abnormal lipid levels for males and females as an LDL-C greater than or equal to 130 mg/dL (3.3 mmol/L), an HDL-C less than 40 mg/dL (1.03 mmol/L), non-HDL-C greater than 145 mg/dL (3.75 mmol/L), and a triglyceride level greater than or equal to 150 mg/dL (1.69 mmol/L). Race/ethnicity was self-reported and categorized as White, Black, Hispanic, Asian, other, and unknown. BMI was categorized as underweight (BMI <5th percentile), normal (BMI 5th – 84th percentile), overweight (BMI ≥ 85th to 94th percentile), and obese (BMI ≥95th percentile) for age and sex in patients younger than 18 years old. For young adults ages 18–25 years old we used absolute BMI cutoffs of normal (BMI < 25 kg/m^2^), overweight (25 – < 30 kg/m^2^), and obese (≥30 kg/m^2^). Corresponding BMI categories were then combined for those younger and older than 18 years of age.

#### Baseline analyses

We tested for differences in lipid parameters (LDL-C, HDL-C, non-HDL-C, and natural log-transformed triglyceride level [lnTG]) in relation to payer-type by conducting multivariable adjusted linear regression models with the baseline lipid value as the dependent variable, payer-type as the independent variable of interest and adjusting for age, sex, and race/ethnicity. We ran a secondary model that additionally adjusted for BMI category (in addition to age, sex, and race/ethnicity) to determine if any differences in dyslipidemia based on payer-type could be attributed to differences in adiposity between payer groups. Finally, we ran separate models that additionally included an interaction term for payer-type and one of the covariates (age, sex, race/ethnicity, and BMI category). By adding the interaction term, we tested if the relationship between payer-type and lipid values was modified by the patient’s age, sex, race/ethnicity, or obesity/overweight status.

#### Changes in lipid parameters during lifestyle counseling

To compare the change in lipid values in response to treatment over time by payer-type, we conducted pairwise multivariable adjusted linear regression models. The dependent variable was the difference in the lipid parameter at baseline to each follow-up visit in separate models (i.e., differences in LDL-C from baseline to first follow up visit). We separately examined change from baseline to first through third visit, as the sample size became too small for comparisons beyond the third follow-up visit. As the time between visits was not consistent for each patient, we adjusted for the length of time between visits. Payer-type was the independent variable of interest, and the model was adjusted for age, sex, race/ethnicity, baseline lipid value, and days between baseline and follow-up visits. The pairwise analyses were restricted to patients with abnormal LDL-C, HDL-C, non-HDL-C, and triglycerides levels at the initial visit for the respective models for that lipid parameter. In the models assessing the change in lipid values between visits, we additionally tested for effect modification by age, sex, and race/ethnicity, and secondary adjustment for BMI category, as described for baseline analysis.

Statistical analyses were conducted with R statistical software (version 3.4.3) and a two-sided *p*-value < 0.05 was considered significant.

## Results

Among the 2487 patients with 8233 clinical encounters over the five-and-a-half-year observation period, 1739 patients (3609 clinical encounters) were eligible for inclusion in the study. Patients were excluded if they were over the age of 25 years (6 patients), did not have a baseline visit in the observation period (593 patients), or were on lipid lowering therapy during the observation period (149 patients). There were 858 (49%) patients, 464 (27%) patients, and 241 (14%) patients that had at least 1, 2, and 3 follow up visits, respectively. Follow up was similar regardless of payer-type (Table 1). There was no difference between the two payer groups in the percentage of patients who had one follow up visit (*p* = 0.8), two follow up visits (*p* = 0.5), or three follow up visits (*p* = 0.2). The median (IQR) time between baseline and first, second, and third follow-up visits were 3.9 (2.8), 9.2 (8.1), and 17.9 (11.9) months, respectively. Of the eligible patients at the baseline visit, 864 (50%) had an abnormal LDL-C (> 130 mg/dL), 554 (32%) had an abnormal HDL-C (< 40 mg/dL), 1061 (61%) had an abnormal non-HDL-C (> 145 mg/dL), and 657 (38%) had an abnormal triglyceride level (> 150 mg/dL). Baseline characteristics of the government-sponsored and private insurance groups are presented in Table 2.

**Table 1:** Number of patients with follow up visits by payer-type

	Government	Private	*P*-value
1st follow up visit, n (%)	172 (49)	686 (50)	0.8
2nd follow up visit, n (%)	89 (25)	375 (27)	0.5
3rd follow up visit, n (%)	57 (16)	184 (13)	0.2

Data presented are number of patients with follow up visits and the percentage of patients with a baseline visit. Government, government-sponsored health coverage; Private, private health insurance

**Table 2:** Baseline characteristics of study population by payer-type

	All patients	Government	Private	*P* value
Total (%)	1739	354 (20)	1385 (80)	
Age (years)	13.0 (4.3)	12.8 (4)	13.0 (4.4)	0.42
Males (%)	843 (48)	174 (49)	669 (48)	0.82
Females (%)	896 (52)	180 (51)	716 (52)	0.82
Race/Ethnicity (%)				
Asian	73 (4)	15 (4)	58 (4)	< 0.001
Black	90 (5)	30 (9)	60 (4)	
Hispanic	121 (7)	59 (17)	62 (5)	
Other	141 (8)	54 (15)	87 (6)	
Unknown	293 (17)	76 (22)	215 (16)	
White	1021 (59)	118 (34)	903 (65)	
Overweight and Obese* (%)	1018 (60)	249 (72)	769 (57)	< 0.001

Data are presented as mean and (SD) *Overweight is defined as a body mass index >85th percentile for age and gender in patients age 2–19 and greater than equal to 25 kg/m^2^ for those 20 years and older. Government, government-sponsored health coverage; Private, private insurance

### Payer-type and baseline dyslipidemia

In age, sex, and race/ethnicity-adjusted models, patients with government-sponsored health coverage were found to have a baseline HDL-C that was 3.5 (1.0) mg/dL lower than patients with private insurance (*p* < 0.001). Patients with government-sponsored health coverage were also found to have higher baseline lnTG (+ 0.14 [0.04], *p* < 0.001), which equates to a triglyceride concentration of 1.15 times higher with government-sponsored care as compared to private insurance. However, the association of government-sponsored health insurance with lower HDL-C and higher lnTG was no longer present after adjusting for BMI category (− 1.1 [0.9], *p* = 0.13, and + 0.05 [0.04], *p* = 0.2 for HDL-C and lnTG, respectively). There was no difference in baseline LDL-C between patients with government-sponsored health coverage and private insurance (+ 3.4 mg/dL [3.0], *p* = 0.3). We did not detect effect modification by age, sex, race/ethnicity, or obesity/overweight status on the relationship between payer-type and baseline lipid parameter.

### Lipid parameter outcomes in response to lifestyle modifications by payer-type

**Fig. 1:**
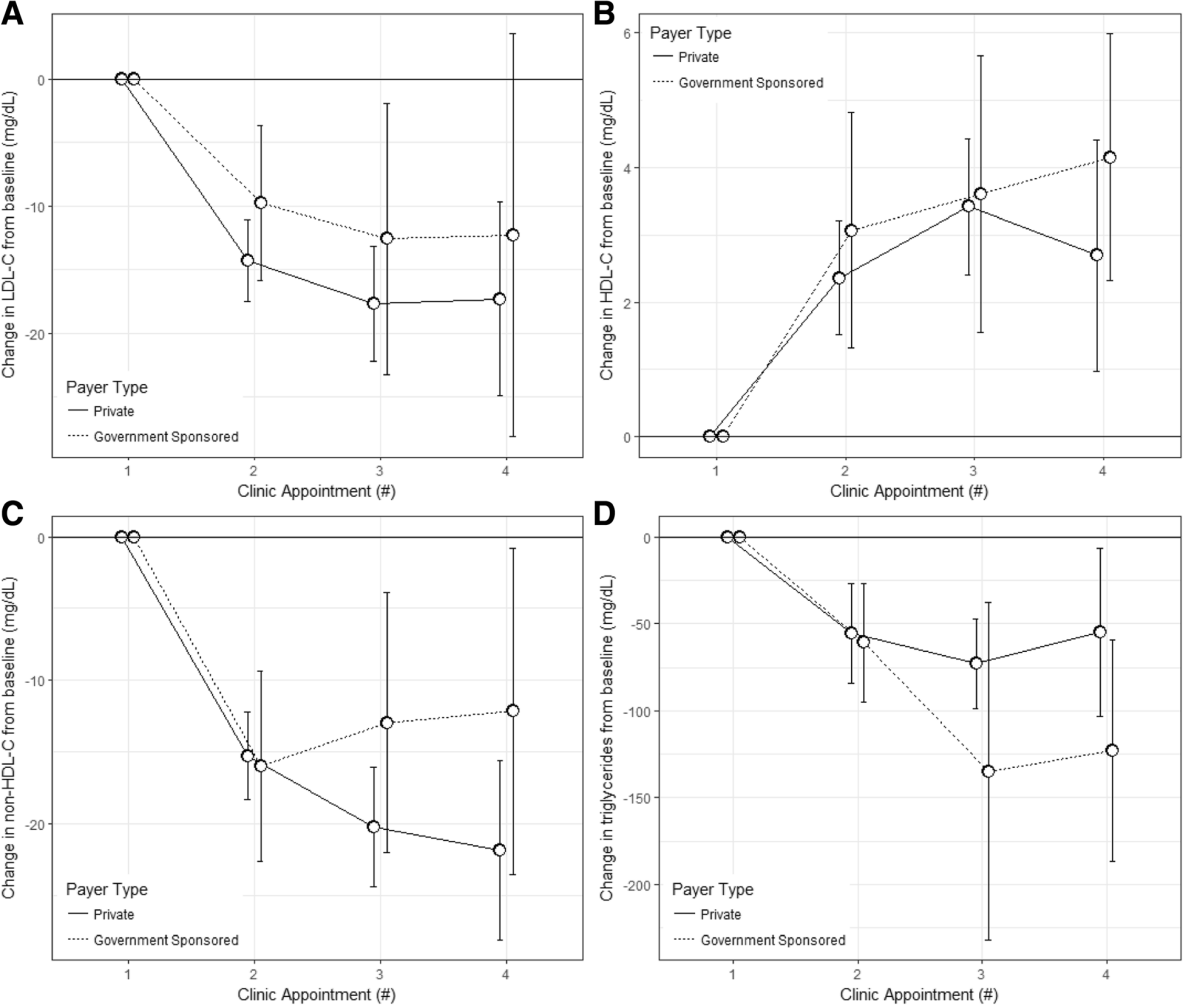
Change in lipid parameters from baseline through the third follow-up visit among those with lipid-specific abnormal levels at baseline and stratified by health insurance payer type. **a**) Mean change in LDL-C (mg/dL) from baseline through the third follow-up visit; **b**) Mean change in HDL-C (mg/dL) from baseline through the third follow-up visit; **c**) Mean change in non-HDL-C (mg/dL) from baseline through the third follow-up visit; **d**) Mean change in triglyceride level (mg/dL) from baseline through the third follow-up visit. All data presented are mean and 95% confidence intervals. HDL-C, high density lipoprotein cholesterol; LDL-C, low density lipoprotein

**Fig. 2:**
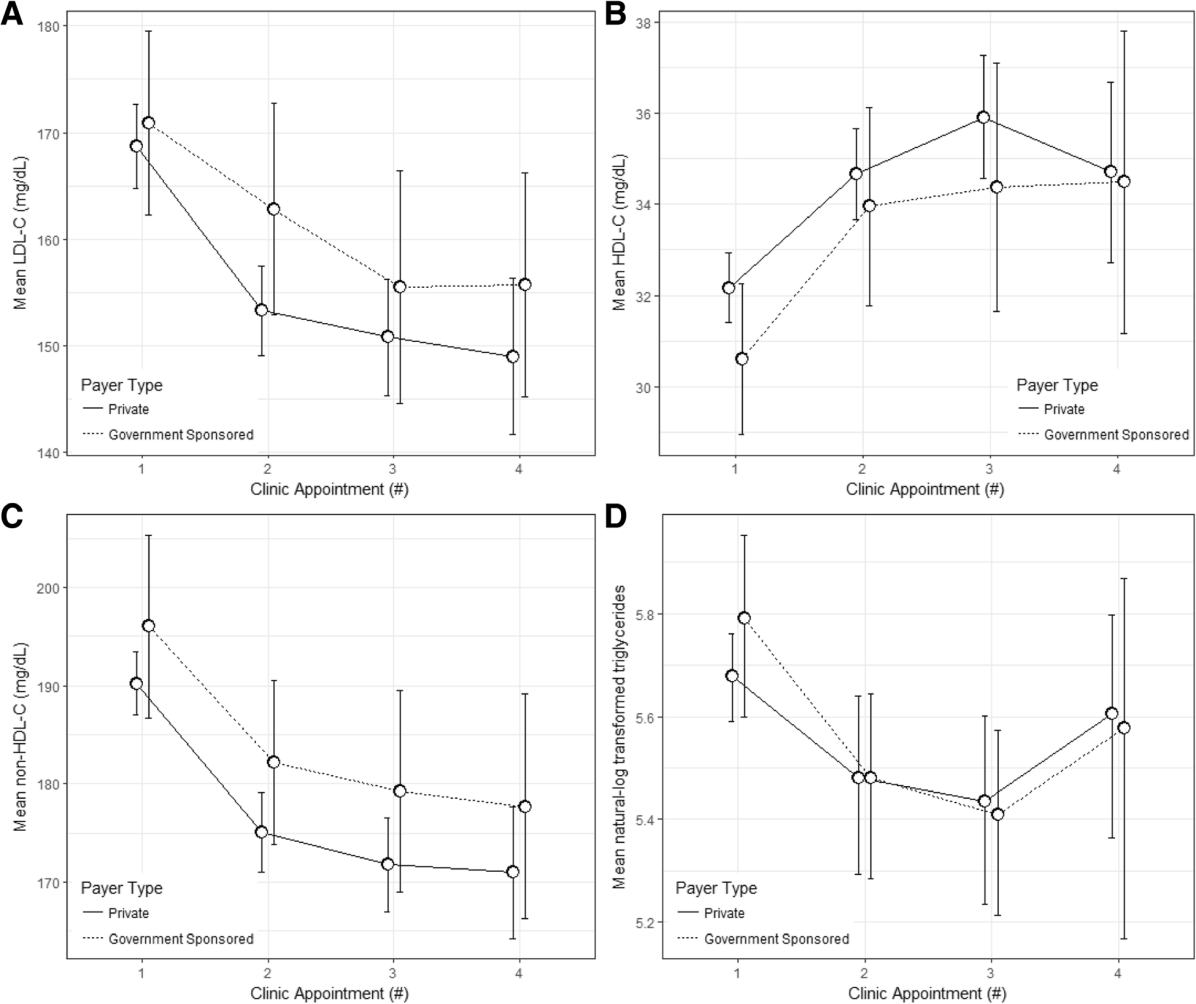
Lipid levels from those with lipid-specific abnormal levels at baseline through the third follow-up visit and stratified by health insurance payer type. **a**) Mean LDL-C (mg/dL) at baseline through the third follow-up visit; **b**) Mean HDL-C (mg/dL) at baseline through the third follow-up visit; **c**) Mean non-HDL-C (mg/dL) at baseline through the third follow-up visit; **d**) Mean triglyceride level (mg/dL) at baseline through the third follow-up visit. All data presented are mean and 95% confidence intervals. HDL-C, high density lipoprotein cholesterol; LDL-C, low density lipoprotein

**Fig. 3:**
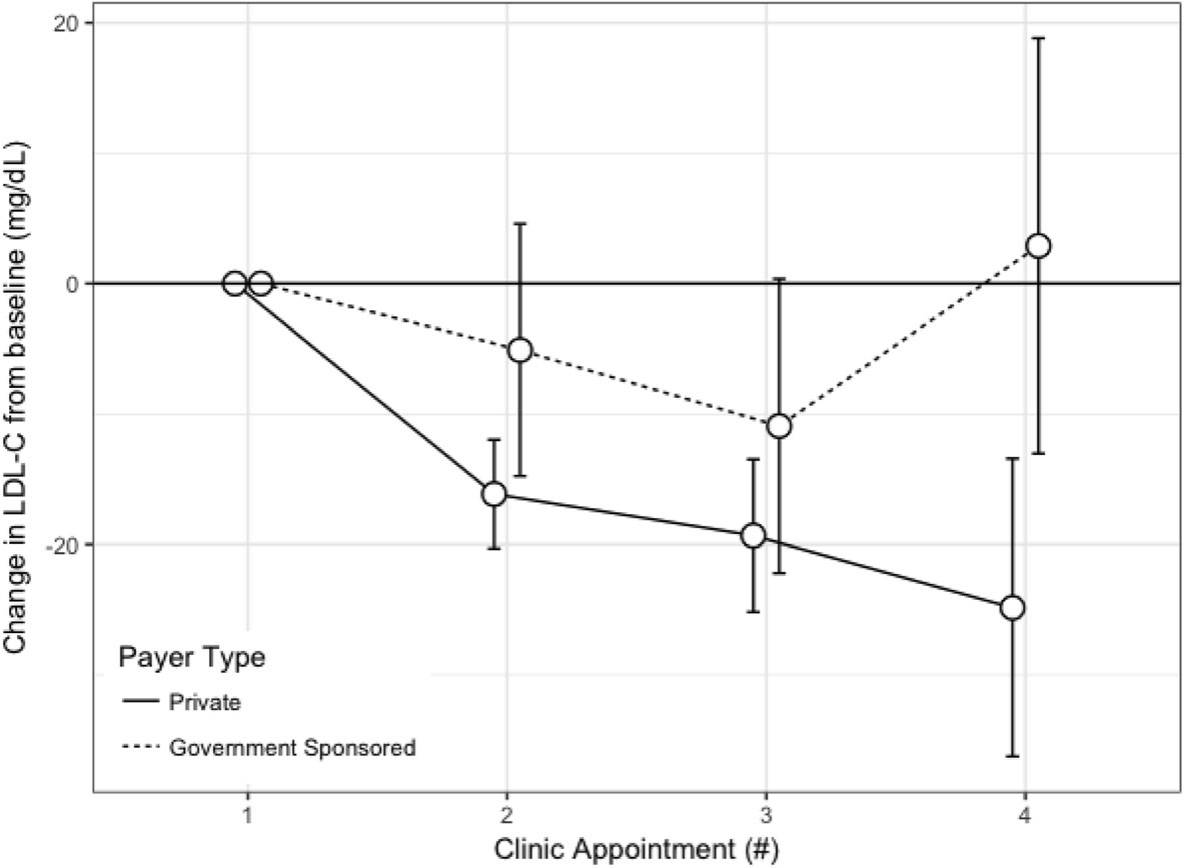
Change in LDL-C (mg/dL) at baseline through the third follow-up visit among patients with self-reported White race/ethnicity and abnormal LDL-C at baseline and stratified by health coverage payer-type. Data presented are mean and 95% confidence intervals. CI, confidence interval; LDL-C, low density lipoprotein cholesterol

The lipid values from the baseline visit through the third follow-up visit are presented in Table 3. Overall, for patients with baseline abnormal lipid-specific values, from the baseline to third follow-up visit, there was a 15 (39) mg/dL decrease in LDL-C, a 3 [7] mg/dL increase in HDL-C, a 20 (37) mg/dL decrease in non-HDL-C, and a 69 (222) mg/dL decrease in triglyceride levels. We observed no association between payer-type and change in lipid values from baseline to first through third follow-up visit, adjusting for age, sex, race/ethnicity, and time between visits (Figs. 1 and 2). Despite no main effect of an association between payer-type and change in LDL-C between visits, we did detect an interaction between patients with self-reported White race and the relationship between payer-type and change in LDL-C (*p* = 0.03). In race-stratified models (White only, abnormal LDL-C at baseline with at least 1 follow-up, *n* = 248), White patients with private insurance had a 12.8 (5.5) mg/dL (*p* = 0.02) additional decrease in LDL-C between baseline and first follow-up as compared to White patients with government-sponsored health coverage, after adjusting for age, sex, time between visits, and baseline LDL-C (Fig. 3). The association was unchanged after additionally adjusting for BMI category. There was no observed association between payer-type and change in LDL-C from baseline to first follow-up in the other race/ethnic groups, although the sample sizes were smaller limiting our power to detect a difference (*n* = 25, 29, 23, and 69 for Black, Hispanic, other, and unknown, respectively). There was no observed effect modification by sex, age, and BMI category on the relationship between payer-type and change in lipid values.

**Table 3:** Lipid parameters at baseline through three follow-up visits among those with abnormal lipid-specific levels at baseline, and stratified health insurance by payer-type

	Baseline				Follow up 1				Follow up 2				Follow up 3			
	n	Government	n	Private	n	Government	n	Private	n	Government	n	Private	n	Government	n	Private
LDL-C (mg/dL)	96	170 (45)	373	167 (38)	87	163 (47)	355	153 (41)	51	156 (40)	202	151 (39)	28	156 (28)	96	149 (37)
HDL-C (mg/dL)	63	31 (6)	233	32 (6)	55	34 (8)	222	35 (8)	30	34 (8)	129	34 (8)	22	35 (8)	69	35 (8)
non-HDL-C (mg/dL)	116	193 (45)	482	191 (37)	108	179 (42)	460	175 (44)	65	179 (42)	253	172 (39)	40	178 (37)	132	171 (39)
TG (mg/dL)*	65	239 (232)	305	222 (122)	59	189 (140)	293	182 (121)	33	196 (70)	163	171 (129)	23	218 (176)	90	190 (129)

Data presented as mean and (SD). *Triglycerides are presented as median and interquartile range. Private, private health insurance; government, government-sponsored health coverage; *LDL-C* Low density lipoprotein cholesterol, *HDL-C* High density lipoprotein cholesterol, *TG* Triglycerides

## Discussion

We present the relationship between payer-type and LDL-C, HDL-C, and triglycerides at baseline and over nearly a six-year period in more than 1700 pediatric patients using prospectively collected standardized data in a large multidisciplinary pediatric lipid clinic. We found that patients with government-sponsored health coverage presented with higher triglycerides and lower HDL-C as compared to those with private insurance. However, the difference in HDL-C and TG is largely attributed to higher rates of overweight and obesity in the government-sponsored care group. There was substantial improvement in lipid profile regardless of payer-type, but White patients with private insurance had additional improvement in LDL-C as compared to White patients with government-sponsored coverage.

In our original hypothesis, we expected that baseline lipid profiles would be associated with payer-type as this is consistent with previous studies in adult and pediatric cohorts examining the association of SES with health outcomes [20, 21]. Although the rise in obesity rates has occurred across all demographic groups, there has been a disproportionate rise in obesity in those of lower SES. In fact, there is evidence that those enrolled in the Medicaid program have higher rates of obesity than those without health insurance and those with private insurance [23]. The increase in obesity has been linked to a variety of potential culprits, including the reduction of the costs of food production, increased number of restaurants, and a shift to jobs that require less physical activity, increased sedentary time, increased consumption of sugar-sweetened beverages, and increased amounts of calorically-dense, nutritional poor foods [22]. In addition, decreased access to recreational activities and healthy foods disproportionately affect those of lower SES due to lack of fresh foods, crime, unsafe built environments, and limited transportation options [24–27].

In order to reduce these health disparities in SES, efforts recently have been made to increase access to health coverage, a modifiable component of SES. As seen in our study, health coverage may allow access to needed preventive care that can slow, or even reverse, the progression of chronic diseases, such as dyslipidemia. This finding is a pleasant surprise as evidence suggests [28] that health coverage alone is rarely sufficient to lead to improvements in care for those of lower SES as it does not address other factors that limit access to care. For instance, in a government-sponsored study in Oregon, there was no evidence that new adult enrollees in government-sponsored healthcare had improvement in their health, despite increased access to health care and increased utilization of services [28]. This is consistent with the seminal RAND insurance study demonstrating that more generous insurance plans led to more health care utilization, but not improved health outcomes [29].

We found that patients who self-reported as White and having private insurance had a greater improvement in their LDL-C as compared to those with government-sponsored coverage. Although we were not able to test this in our data because self-reported income levels were not available to us, patients who reported themselves as White and had private insurance may have additional financial resources that enabled them to improve their LDL-C through more expensive lifestyle changes (e.g., enrollment in costly sports programs, leaner meats, fish and vegetables). In contrast, improvements in triglycerides, which did not differ by payer-type, may be driven by less expensive dietary choices, such replacing sugar-sweetened beverages with water or reducing overall portion sizes.

The use by this subspecialty clinic of a standardized approach to lifestyle modification counseling, the SCAMP, may have mitigated differences in changes in lipid levels based on payer-type. One potential explanation is the consistent approach guided by the SCAMP that all providers in the clinic used to guide assessment of risk factors and counseling of patients. The importance of a stable access to specialists is consistent with a Commonwealth Fund study of adults enrolled in Medicaid that found that those with continuous coverage over the past year had meaningful improvements in self-reported health compared to those who only had Medicaid coverage intermittently over the preceding year [30]. Similarly, in a study conducted by the CDC, one of the most important factors distinguishing health outcomes between those living in an urban area and their semi-rural counterparts was having a consistent provider, even when medical services are less available [31].

### Limitations

The strengths of our study include prospectively collected data and a relatively large pediatric cohort. However, this study also has limitations. First, the type of health coverage captures only one aspect of SES [14, 32]; parental education level and self-reported incomes are known to be important SES measures that were not available in the dataset. Second, we may not have had sufficient power to detect an overall difference in response to treatment as we had a small number of patients with government-sponsored coverage. However, if this were the case in our data, the undetected effects would likely not be of a clinical significance. Patients self-classification of race/ethnicity was frequently incomplete. Similarly, a progressively decreasing sample size, prevented us from making a valid conclusion in regards to longer-term outcomes beyond three visits. Our study also is limited by the inability to control for a number of factors that could potentially effect behavioral change, including family history and smoking history. In our study, we used payer-type as a marker of socioeconomic status; other markers were not available to us. Another limitation was that providers had access to the patient’s health insurance type at the visit and it cannot be guaranteed that this information did not influence patient counseling. As the population evaluated in this study was derived from a referral population, the patients may have been more motivated than the general population to make lifestyle changes and may have had more resources to make lifestyle changes. For instance, patients in our population have already demonstrated their engagement by visiting a preventive cardiology clinic.

## Conclusions

Overall, our findings suggest that pediatric patients with government-sponsored health coverage along with myriad potential concomitant factors that accompany this type of coverage, such as low socioeconomic status and decreased access to certain resources: 1) present more frequently with high triglycerides and low HDL cholesterol as compared to those with private insurance, although this is largely explained by higher BMI and obesity in the government-sponsored payer group; 2) changes in lipid outcomes while attending a well-resourced, multidisciplinary clinic are similar overall between the two groups; and 3) subgroup analysis suggests that pediatric patients with self-reported White race with private insurance had more improvement in LDL cholesterol over time as compared with White patients with government-sponsored coverage.

## Data Availability

The data that support the findings of this study are not publicly available due to privacy concerns as they were derived from an internal quality improvement project; limited de-identified data may be requested from the corresponding author (JH).

## Abbreviations

BMI: Body mass index
CDC: Centers for Disease Control
CVD: Cardiovascular disease
HDL-C: High density lipoprotein cholesterol
LDL-C: Low density lipoprotein cholesterol
QI: Quality improvement
SCAMP: Standardized Clinical Assessment and Management Plan
SES: Socioeconomic status
